# The Current View on the Paradox of Pain in Autism Spectrum Disorders

**DOI:** 10.3389/fpsyt.2022.910824

**Published:** 2022-07-22

**Authors:** Olena V. Bogdanova, Volodymyr B. Bogdanov, Adrien Pizano, Manuel Bouvard, Jean-Rene Cazalets, Nicholas Mellen, Anouck Amestoy

**Affiliations:** ^1^CNRS, Aquitaine Institute for Cognitive and Integrative Neuroscience, INCIA, UMR 5287, Université de Bordeaux, Bordeaux, France; ^2^Laboratoire EA 4136 – Handicap Activité Cognition Santé HACS, Collège Science de la Sante, Institut Universitaire des Sciences de la Réadaptation, Université de Bordeaux, Bordeaux, France; ^3^Centre Hospitalier Charles-Perrens, Pôle Universitaire de Psychiatrie de l’Enfant et de l’Adolescent, Bordeaux, France; ^4^Department of Neurology, University of Louisville, Louisville, KY, United States

**Keywords:** pain, autism, perception, reaction, evaluation, expression

## Abstract

Autism spectrum disorder (ASD) is a neurodevelopmental disorder, which affects 1 in 44 children and may cause severe disabilities. Besides socio-communicational difficulties and repetitive behaviors, ASD also presents as atypical sensorimotor function and pain reactivity. While chronic pain is a frequent co-morbidity in autism, pain management in this population is often insufficient because of difficulties in pain evaluation, worsening their prognosis and perhaps driving higher mortality rates. Previous observations have tended to oversimplify the experience of pain in autism as being insensitive to painful stimuli. Various findings in the past 15 years have challenged and complicated this dogma. However, a relatively small number of studies investigates the physiological correlates of pain reactivity in ASD. We explore the possibility that atypical pain perception in people with ASD is mediated by alterations in pain perception, transmission, expression and modulation, and through interactions between these processes. These complex interactions may account for the great variability and sometimes contradictory findings from the studies. A growing body of evidence is challenging the idea of alterations in pain processing in ASD due to a single factor, and calls for an integrative view. We propose a model of the pain cycle that includes the interplay between the molecular and neurophysiological pathways of pain processing and it conscious appraisal that may interfere with pain reactivity and coping in autism. The role of social factors in pain-induced response is also discussed. Pain assessment in clinical care is mostly based on subjective rather than objective measures. This review clarifies the strong need for a consistent methodology, and describes innovative tools to cope with the heterogeneity of pain expression in ASD, enabling individualized assessment. Multiple measures, including self-reporting, informant reporting, clinician-assessed, and purely physiological metrics may provide more consistent results. An integrative view on the regulation of the pain cycle offers a more robust framework to characterize the experience of pain in autism.

## Introduction

“*On the one hand, some people with autism can tolerate extreme heat, cold or pressure and seem relatively insensitive to pain. On the other hand, they may experience intense pain from idiosyncratic sources but struggle to communicate it*.” Citation from Spectrum Autism Research News (1).

Autism Spectrum Disorder (ASD) is a neurodevelopmental disorder, affecting around one birth out of 150 worldwide (2). According to new report from Centers for Disease Control and Prevention, the incidence is as high as 1 in 44 children, leading to a prevalence estimated at 2.3% (3), a male-to-female ratio around 3:1 (4). ASD significantly decreases the person’s educational, social and employment opportunities. Among other comorbidities, pain is frequent, sometimes undiagnosed or diagnosed with delay in children with ASD (5–8). Children with autism are about twice as likely as their typical peers to experience chronic or recurrent pain (9). Various co-morbidities in ASD, such as epilepsy (10, 11), joint hypermobility-related disorders (12), gastrointestinal disorders (13), anxiety (14), and sleep problems (15) may be additive sources of pain. Individuals with ASD face significant inequities in healthcare, despite their high rate of medical comorbidities. They tend to have poorer health outcomes (16) and higher mortality rates (10, 17, 18) than their neurotypical peers. Aside from other core symptoms, such as socio-communicational difficulties, repetitive activities and/or interests and atypical sensory modulation, some individuals may present high levels of Self-Injury Behaviors (SIB; 19) which may be related to pain reactivity in ASD (20, 21).

The paradox of pain perception in ASD has attracted the attention of researchers for many years. Atypical pain perception, expression and difficulties in pain assessment in people with ASD have been described (22–27). On one hand, some studies report a decrease or absence of pain reactivity in daily life mostly based on reports from self or others and clinical observations (22, 28). On the other hand, various results show equal or greater responsiveness to painful stimuli in experimental conditions (29–31). Atypical pain sensations, like allodynia (extreme sensitivity to usual non-painful tactile stimulation causing intensive pain), paradoxical heat sensation (gentle cooling perceived as hot or burning), and hypoesthesia (decreased pain sensitivity) are also reported for ASD (32). Various hypotheses attempted to explain observed alterations. However, none of these hypothesis by themselves were able to reconcile these apparently contradictory findings.

The International Association for the Study of Pain defines pain as “an unpleasant sensory and emotional experience associated with, or *resembling* that associated with, actual or potential tissue damage” (33). On one hand, pain is a universal “alert sign,” considered to be an instinct to prevent chronic and repetitive injuries. On the other hand, pain is a subjective, personal, and multifaceted construction that may be modulated by various biological, psychological, and social (cultural and contextual) factors. It may be provoked by physical, emotional, or social triggers (e.g., evoked by the observation of the suffering of others; 34). Pain regulation is essential for successful adaptation to life events, development of social interactions skills and empathy (35, 36) and has been implicated in social difficulties in autism (37).

The physiological mechanisms and both peripheral and central pathways of pain processing are well documented. Following activation of specific nociceptors, pain signals are transmitted *via* the peripheral nervous system (PNS) to the central nervous system (CNS), composed of the spinal cord and the brain. The CNS is responsible for processing, integrating, and interpreting the information sent from the PNS, ultimately elaborating into a complex sensory experience (Supplementary Table 1). The subcortical and cortical centers in the brain coordinate all the motor responses produced to diminish or avoid painful input. Both thresholds and intensity of pain perception may be modulated by internal mechanisms. Endogenous pain modulation includes the “***endogenous analgesia*****”** which refers to the pain-inhibiting pathway originating in the brainstem and terminating in the spinal cord. Another mechanism, “***descending pain modulation system,”*** consists of a large network in the brain which regulates pain sensory input to the CNS and behavioral reactivity. The internal mechanisms can change the meaning of pain based in previous exposure and pain expectancy, its influence on our emotional state, and its relevance to our survival. However, these processes may be affected by mood, neurological disorders, environmental and genetic factors (36, 38, 39).

**FIGURE 1:**
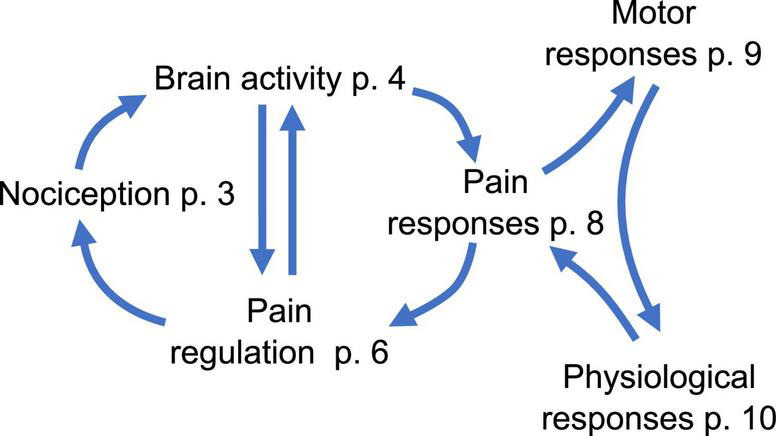
The simplified view of the cycle of pain (for illustrative purposes) with the numbers of corresponding pages of the paper.

Whatever the medical condition or the noxious input, individuals can use a large number of cognitive or behavioral strategies for coping with pain. These reactions include cognitive self-instruction; visual imagery and distraction; body relaxation training; seeking for social support or, alternatively, withdrawing from social contact; worrying or even catastrophizing; each of which can either decrease or increase pain sensation (40). Therefore, pain perception and pain responses form dynamic feedback loops (41–43). Interruptions in this “pain cycle” (Figure 1) may cause long-term plasticity in pain perception and regulation mechanisms which can be responsible for increased or decreased pain sensitivity, or even allodynia (44).

When one considers the perspectives of pain research in ASD, one may ask what would be the origins of altered pain sensitivity and reactivity in ASD? In other words, ***where*** and ***when*** did the circle of pain start to go wrong, and for ***whom***? Another important question, **what** should be done to overcome that issue?

To address this, we will review in the second section, the recent literature on pain processing and regulation in individuals with ASD, from initial stages to brain response in the “*pain matrix*.” We will propose that altered cognitive and emotional control of pain may also contribute to the altered pain response in ASD, in line with the chronic pain disorders hypothesis (42).

In the third section, pain responses in individuals with ASD and clinical methods for pain assessment (self-reports and reports by others, physiological measures, body responses) will be synthesized, highlighting the need for objective and multimodal approaches. The significance of an individualized approach for pain assessment and management will be highlighted.

Thereafter, the altered pathophysiological pathways and brain circuits involved in the emergence of ASD will be reviewed and discussed with regard to their possible involvement in the pain regulation. We will conclude by proposing an integrative view of the pain cycle, which, along with existing mechanisms of pain alterations in ASD, defines key targets for further pain research in ASD.

***Introduction Highlights.** Individuals with ASD demonstrate a wide range of alterations in pain reactivity. However, the question of pain perception in autism is understudied. Processing of pain includes several stages with feed-back and feed-forward interactions, forming “a pain modulation circle.” Reactivity to and coping with pain in autism may be altered because of various interruptions of this cycle; these can be configured differently in each individual with ASD.*

## Neurophysiological Processing of Pain in Autism Spectrum Disorder

### Initial Stages of Pain Signaling: From Nociceptors to the Central Axis

While pain perception arises from the combination of different somatosensory modalities (45), processing of a painful stimulus starts from nociceptors (Supplementary Table 1), which are specialized for perception of painful stimuli of different modalities, detecting potential thermal, mechanical, and chemical tissue damage. The signal is transmitted to the CNS *via* more rapid, myelinated Aδ (A-delta) fiber axons responsible for acute pain transmission and slow, non-myelinated C-fibers (small fibers). The combination of these signals creates two phases of pain perception; fast and intense, followed by diffuse and dull pain (36). In individuals with ASD, atypical small fiber density was recently described (46, 47). These alterations were associated with autistic symptoms, and may also contribute to hypoesthesia (already explained above) and allodynia in autism (47). However, increased pain and touch sensitivity were observed specifically in areas innervated by C-fibers, providing a candidate mechanism for atypical avoiding behaviors or hypersensitivity in ASD. Another hypothesis could be a pathological activation of silent nociceptors, which are normally unresponsive to noxious stimuli, but become responsive to mechanical stimulation (30).

The initial stages of noxious signaling are usually characterized by the so-called nociceptive “thresholds,” i.e., the lowest intensity at which noxious stimuli are perceived to be painful. The thresholds may be different depending on whether noxious stimuli are thermal, mechanical, or due to compression. Studies in individuals with ASD have reported contradictory findings on pain thresholds depending of the type of stimuli (24, 25, 29, 31, 32, 48). No difference between neurotypical subjects and individuals with ASD was reported for electrocutaneous pain (48) and thermal pain (14, 49) thresholds. Lower thresholds (i.e., higher sensitivity) were observed for pressure-induced pain (29, 30) and heat-induced pain (31). By contrast, subjects with ASD displayed hyposensitivity to pinprick-induced mechanical pain (32).

In addition to these inconsistent results, individuals with ASD may display hypersensitivity to stimuli usually considered painless. These responses may be accompanied by absence of reactivity to potentially hazardous and noxious stimuli (1). Such *stimulus overselectivity*, when an individual responds only to a limited category/amount of incoming sensory information, was not confirmed on group level, however, (50). Furthermore, sensory processing patterns are known to change with time, depending on the context in which they are estimated (51). Both hyper- and hyposensitivity to the same type of stimuli may exist in the same person with ASD, challenging the suggestion that only the PNS is implicated in the observed alterations (52). In addition, the results of such psychophysical evaluation of pain detection and discrimination may be confounded by other factors like attentional resources, levels of anxiety or task performance capacities which may be different in autism.

At the level of the spinal cord, complex interactions between excitatory and inhibitory interneurons have been shown to modulate pain signal transduction. According to the “gate control” theory (53), concurrent activation of large sensory afferents from the skin (Aβ-fibers) could suppress transmission in small unmyelinated C-fiber afferents and therefore block pain perception. As a consequence, tactile stimulation of a painful area may relieve pain (54).

The relation between self-stimulation and self-injuring behavior and response to pain in autism has been discussed (20). A study measured SIBs and pain signs in non-verbal individuals with ASD (55) revealed increased behavioral signs of pain in adults with chronic self-injury. That suggests that the SIBs might be a coping strategy to manage chronic pain. Children with ASD showed a significantly reduced pain sensitivity and increased tactile sensitivity after somatosensory- directed therapeutic manipulations (with touch, proprioception, vibration, and stereognosis; 56); this effect was not find in the control group. The authors suggested that repetitive somatosensory distraction, by an increase of affective-motivational input affects pain processing and alleviates pain sensation, in line with findings in various chronic pain conditions (57–59). Although the effects of these therapeutic manipulations may be ascribed to central pain processing and top-down regulation, normalization and integrity of peripheral sensory perception likely also contributes. However, the causal relations between SIB and altered pain perception in ASD is far from understood.

***Initial stages of neurophysiological processing of pain in ASD highlights.*** The evidence for alterations in pain transduction at the level of peripheral receptors in ASD is limited to reports of decreased nociceptor density. That question needs to be more robustly corroborated, given that pain thresholds in individuals with ASD and the control groups weren’t different in most studies. However, peripheral nerve alterations may partly explain sensory dysfunctions like *allodynia* – painful atypical response to touch, and *hypoesthesia* – hyposensitivity to injurious stimuli, but also *paradoxical heat sensation* documented in the battery of sensory tests in autism.

### Atypical Brain Activity for Pain Processing in Autism Spectrum Disorder

#### The Pain Matrix: First Level of Response “The Nociceptive Matrix” (Thalamus, Somatosensory Cortex)

##### The Thalamus

**FIGURE 2:**
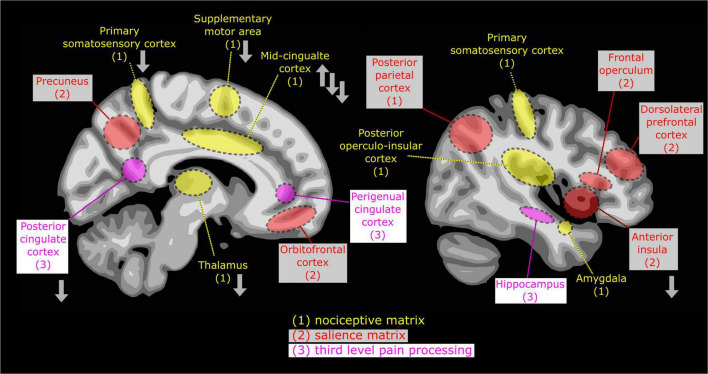
Basic view of ascending pain processing, delineated on two brain sections, with emphasis on early, sub-conscious (*1, “nociceptive matrix*”: the posterior operculo-insular cortex, the primary sensory areas, p-mid-cingulate cortex, supplementary motor area and the amygdala) and conscious pain perception (2, “sa*lience matrix*”: the anterior cingulate cortex, the anterior insula, posterior parietal, prefrontal and orbitofrontal cortices), and (3, “*areas of third-order brain activation*”: the hippocampus and the anterior and posterior cingulate). Nociceptive cortical processing is initiated in parallel in sensory, motor and limbic areas. Some activation may last longer than voluntary motor reaction. Based on brain response dynamics and models described in Bushnell et al. (42), Garcia-Larrea and Bastuji (70). The alterations in pain-induced responses in the brain of individuals with ASD are shown with gray arrows according to Failla et al. (62), Chien et al. (71), and Gu et al. (72).

The thalamus, a regulatory hub for sensory inputs from different modalities, is involved in pain processing (60). It can be reorganized following chronic pain, altering maps of noxious and innocuous stimulation and leading to the perception of innocuous stimuli as nociceptive (61). In individuals with ASD, both structural and functional deviations in the thalamus have been found (62–66; Figure 2). The available studies on the resting state or anatomical connectivity yielded heterogeneous results. Both hypo-(64, 67) and hyperconnectivity (65, 68) between thalamus and cortical areas were demonstrated in ASD. During an aversive sensory stimulation, thalamocortical connectivity was attenuated, while connectivity between thalamus and subcortical areas (putamen, hippocampus, and amygdala) was, in contrast, enhanced in individuals with ASD when compared to controls (69). This hyper-connectivity in response to aversive stimuli could indicate a lack of cortical inhibition. Additionally, connectivity between thalamus and amygdala is thought to play a role in directing attention to emotionally salient information. This suggests that modified thalamus connectivity may be responsible for over-attribution of “pain” salience to benign tactile stimuli (allodynia).

In a recent study investigating different stages of pain-induced brain responses, Failla et al. (62) showed that processing of noxious stimuli in the brain differs between subjects with ASD and neurotypicals, with regard to the duration and type of pain. In this study, adults with ASD showed significantly less brain activation in the thalamus compared with controls during sustained pain stimulation (62). In contrast, acute brain responses to pain were similar across groups. That reorganization of thalamocortical and thalamosubcortical pain-processing pathways may be responsible for reduced pain awareness due to attenuated thalamocortical signaling. In addition, increased unspecified distress due to upregulated signaling between thalamus and sub-cortical structures noxious may result in possible perception of neutral stimuli as in ASD.

##### The Somatosensory Cortex

The functional and structural modifications in one of the earliest brain areas activated by noxious stimuli, the primary somatosensory cortex, S1 have been detected in subjects with ASD (73, 74) along with altered sensitivity profiles (32, 75). In addition, reduced gray matter volume in somatosensory brain areas and possible disruptions in thalamocortical white matter fiber pathways were associated with higher SIB scores in autism (76). In the above-mentioned study (62) sustained painful stimulation (heat stimuli administered to a right lateral calf), was associated with significantly less activation in the left S1 and bilateral secondary somatosensory cortices S2 in the group of participants with ASD than in their neurotypical peers. Again, brain activation during the initial stage of stimulation was not different across these groups.

#### The Pain Matrix: Salient Encoding of Pain Stimuli “The Salience Matrix” (The Cingulate Cortex, Prefrontral Cortex and Insula), and Their Interactions With Third Order Areas

***Activation of “salience matrix***” regions is not specific to painful stimulation. These circuits may be activated by any sensory stimulus that rises above the sensory threshold. Such co-activation adds the “salience” meaning of noxious stimuli and triggers attentional control of pain-evoked response (70). The coordinated response within the sensory-specific and second-order networks (Supplementary Table 1 and Figure 2) is crucial to create links between pre-conscious nociception and conscious pain (36, 43).

***The cingulate cortex:*** Besides its role in the attentional component of pain stimulus processing (Supplementary Table 1), the cingulate cortex plays a role in the brain response related to negative affect and goal-directed behavior, resulting in “urgency to act” (77). The alterations in cingulate cortex function have already been documented in ASD (78, 79). Decreased engagement of the anterior cingulate cortex in participants with autism was also observed in the above-mentioned study of sustained pain responses (62). In addition, during a heat-induced pain, both amplitudes and latencies of evoked potentials in the cingulate gyrus were found to be lower in adults with ASD compared to controls, while pain scores were not significantly different between groups (71). Such results suggest that the cingulate cortex seems to be less implicated in general, but may have a specific role at the latest stages of pain processing in ASD, contributing to the motor hyporesponsiveness of individuals with autism.

***The insula*** is involved both in the initial perceptual stage and in the cognitive encoding of pain (80). Structural and functional changes in the insula, as well as alterations in connectivity, were observed in autism, as reviewed in Caria and de Falco (81), Uddin and Menon (82). Decreased insula activation in participants with autism was also observed during sustained pain (62). The insula is considered as a hub for *interoception*, i.e., representation of our own internal word, (83, 84)*; self-awareness* and *bodily self-consciousness* (85); which have generally been reported as atypical in population with ASD (86–92). Disrupted engagement of the insula during pain processing may be responsible for altered pain awareness and/or cognitive pain representation (49).

***The (pre-)frontal cortex*** participates in top-down cognitive and anti-nociceptive short- and long-term controls over pain perception and reaction (93, 94). Less prefrontal cortex engagement may be related to reduced antinociceptive feedback (Supplementary Table 1 and Figure 2). Even if pain-related prefrontal cortex activation does not seem to differ between individuals with and without ASD (62), it is negatively correlated with levels of perceived pain in participants with ASD. Another recent results reported a reduced prefrontal cortex response to painful stimulation in participants with ASD (95).

The reciprocal connections between *prefrontal cortex, hippocampus, and amygdala* are known to be implicated in different stages of pain processing (96). It has been reported that noxious stimuli modulate prefrontal-hippocampal connectivity and impact behavioral performance (97). Chronic pain causes neuroplastic changes in these regions of the brain, resulting in aversive states and memory deficits (98, 99). In ASD, structural and functional alterations have been observed in the hippocampus-prefrontal cortex-amygdala network (100–102). The interruption of maturation of these brain areas caused by chronic pain in early life has been proposed as a predisposing factor for ASD (103). Taking into account recent theories about reorganization of pain processing pathways during the transition from acute to chronic pain proposed by De Ridder et al. (104), one may speculate that such a reorganization during perinatal development could explain alterations observed in ASD. Further research on this topic may provide a more unified account of how sensory and social peculiarities impact together the perception and processing of pain in subjects with ASD (37).

***Default mode network (DMN)*** may also be related to pain processing in autism. According to recent models (104), chronic pain results in reorganization of pain networks in the brain and also implicates the DMN (*the medial prefrontal cortex, posterior cingulate cortex, inferior parietal cortex and the precuneus*; 105–107). That leads to integration of pain into body schema and changes in its appraisal. Little is known about DMN activity during pain processing in subjects with autism, but numerous alterations of DMN activity and in its connections with other brain areas have been described in subjects with ASD (108).

By referring to the model of chronic pain, we may suggest reorganization of thalamocortical and thalamosubcortical pain-processing pathways in ASD. That plasticity could be partly responsible for reduced pain awareness due to attenuated thalamocortical signaling. Second, reduced activation of the second-order brain areas may lead to deviant pain consciousness and hypo- or delayed activation of structures implicated in goal directed behaviors and motor responses. Finally, increased unspecified distress due to upregulated signaling between thalamus and sub-cortical structures, might lead to perception of neutral stimuli as nociceptive.

***The “pain matrix” in ASD highlights.** Results of neuroimaging studies of pain processing in ASD are limited but suggest atypical brain activation patterns. Decreased activation in most of the areas of the pain matrix, starting from earliest cortical and sub cortical structures, during sustained pain stimulation in contrast to acute pain, supports the hypothesis of “feedback default” or “atypical top-down pain regulation” in ASD. Possible re-organization of pain processing pathways in early stages of development in ASD may be responsible for such alterations. Taken together, available information on central pain processing in ASD may explain the paradoxical dissociation between (near to-) intact low-level perceptive abilities in pain detection tasks and altered salience and awareness of pain and its affective components.*

### Pain Perception Modulation in Autism Spectrum Disorder

#### The Descending Pain Control Pathways

The descending pain modulatory system involves connections between the (pre-)frontal cortex, the thalamus, the periaqueductal gray (PAG) and the *amygdala* (Supplementary Table 1 and Figure 2). Among these brain structures, *the amygdala* appears as a key component of top-down regulation. The amygdala allocates attention and assigns emotional value – either positive or negative – to sensory information, thus leading to adaptive behavioral and affective responses and also contributing to emotional memory. Under short-term aversive conditions, such as stress or fear, amygdala activation induces hypoalgesia. Contrary to this, during chronic pain caused by various medical conditions, long-lasting functional plasticity of amygdala activity is related to enhanced nociceptive responses, including hyperalgesia, aversive behavioral reactions and anxiety-like states (109, 110). The amygdala has received considerable attention in ASD studies (111). For example, the amygdala in individuals with ASD show both microscopic (112) and macroscopic structural regional abnormalities. Recently, in a large sample of 1,571 participants with ASD, increased thickness in the frontal cortex and reduced subcortical amygdala volumes were reported (113) as well as connectivity atypicalities (114). In line with the so-called “weak amygdala’s emotional modulation hypothesis in ASD” (115), such deviant regulation may yield atypical behavioral and social/psychological responses (116). Reduced connectivity between the prefrontal cortex and the amygdala during unpleasant stimulus processing was recently observed in children with ASD (117). That suggests that alterations in the interconnectivity of these structures may play a role in the blunted behavioral responses to pain in autism. Importantly, the amygdala is activated by pain as early as the first-order cortical areas (118). Thus if prefrontal networks are unable to exert inhibitory modulation, it may remain over-activated, producing a cascade of autonomic and behavioral reactions.

The descending pain modulation pathways can be both facilitatory as well as inhibitory. Facilitatory pathways are the ones which enhance pain perception, while inhibitory pathways suppress pain perception. Endogeneous opioids are released at synapses at multiple points in the PNS to block the ascending pain transmission signal. Moreover, during chronic pain, these pathways are plastic, resulting in pain sensitization or other perceptual alterations such as allodynia or nocebo (119). Alterations of the balance between descending controls, both excitatory and inhibitory, are involved in some dysfunctional and chronic pain states such as fibromyalgia (120–122) that has been associated with higher rate of autistic traits (123).

Of the endogenous pain modulatory mechanisms, the Diffuse Noxious Inhibitory Control (DNIC), is essential and often been described as “pain inhibits pain.” That occurs when response to a painful stimulus is inhibited by another, often spatially distant, noxious stimulus. The DNIC is predominantly studied in humans using the psychophysical paradigm of Conditioned Pain Modulation (CPM; 124, 125). The most commonly investigated test stimuli are pressure pain threshold (PPT) and cold water immersion is the most frequently studied conditioning stimulus (126). In ASD, recent results suggested a preserved CPM effect. The measures of PPTs increase significantly after a cold conditioning stimulus in both adults with and without ASD (32, 127). Nevertheless, as the pain scores were very variable in individuals with ASD, with a greater range of extreme scores than in control group, results must be interpreted with caution (127).

#### Anticipation of Painful Stimuli

Brain activity patterns accompanying pain anticipation and processing were studied in high functioning adults with ASD and matched neurotypical individuals (72). Stronger activation in the anterior cingulate cortex was observed during the anticipatory phase in the group of participants with autism, while brain responses during painful stimulation were not different from neurotypical peers, in line with (62). The increased anticipatory brain response was paralleled by augmented behavioral sensitivity to *anticipated (expected)* pain in individuals with autism. This may seem to be contrary to previous observations of decreased response to sustained pain stimuli. This may be explained by the fact that in Gu et al. (72), contrary to Failla et al. (62) a cued paradigm was used to force participants to attend to incoming noxious stimuli and concentrate on the sensory aspects of stimulation. It should be noted that in this study, participants with ASD pre-selected a lower level of painful stimulation than healthy individuals, consistent with their hypothesized nociceptive hypersensitivity. The idea being developed here is that one may explain one of the paradoxes of pain perception in autism: expected pain perception is enhanced due to stronger attentional engagement.

#### Pain Resonance and Empathy

A growing body of evidence suggests that the ability to understand and feel compassion for another’s pain, so called “empathy for pain” (128) is underpinned by the same neural structures that are involved in the direct experience of pain. Observing someone experiencing pain induces brain response in the *insula, anterior cingulate cortex, the brainstem and the cerebellum*, but also emotional and physiological responses similar to those caused by a direct experience of pain (129–131). Empathy for pain is hypothesized to be based on the subject’s own experience of pain. It may be related to vicarious pain perception, the sensation of pain on one’s own body while observing the pain of others (132). Results from studies of vicarious pain perception and empathy for pain in ASD are contradictory. Some studies reported no difference from typical peers in behavioral and neuronal signatures of empathy for pain, while others suggest atypical brain activations and reactions in ASD (29, 109, 133–135).

Observation of someone’s injured hand vs. non-injured hand (from a first-person perspective) induced strong and consistent activations of *cingulate, somatosensory and insular* cortices both in participants in control and ASD groups (109). These responses are accompanied by the same level of pupil dilation, an estimator of physiological arousal. Similarly, no group difference was found in the activation of the *insula* during a task based on painful stimulation applied to both the other’s hand and one’s own (48). Similar results were obtained from the observation of facial expressions of others experiencing painful stimuli (134, 135); both neurotypical subjects and participants with ASD exhibited activation in regions involved in pain processing (*the insula, somatosensory cortex, supplementary motor area, periaqueductal gray, and prefrontal cortex*), in affective reaction (*orbito-frontal cortex, the amygdala*) and in face and body part processing (*fusiform face area, extrastriate body areas*). These results would suggest that typical brain processes involved in shared representations of pain or emotional contagion are intact in autism.

In contrast, other results revealed atypical brain activations and physiological and behavioral responses to vicarious pain in ASD. In the same study cited above (135), videos of someone’s limbs in painful vs. non-painful situations caused less activation in the *inferior frontal gyrus, the precentral gyrus, the frontal orbital cortex and the medial prefrontal cortex* in adults with ASD compared to neurotypical controls. As these regions are known as important parts of motor resonance and empathy for pain network (136) their hyporesponsiveness may contribute to emotional resonance alterations in autism.

In the study of Gu et al. (133), subjects observe images of someone’s hands or feet (from first-person perspective) in painful or non-painful situations. In that study subjects with autism were less able to discern pain in others, but demonstrated higher activation *in the anterior insula and extrastriatal body areas*, and lower activation of right *prefrontal cortex* compared to controls. In addition, participants with ASD had augmented empathetic pain-related skin conductance response. That response correlated with brain activation evoked by images, suggesting higher level of physiological arousal and sympathetic nervous system engagement, compared to control participants.

In general, empathic responses are described as non-typical in ASD (137). Although general empathy and empathy for pain are distinct, they may share common neurological and physiological mechanisms. It has been reported that in subjects with ASD and their neurotypical peers, lower empathy is correlated with greater *anterior cingulate cortex and insula* activation in a pain anticipation task (72). Consistent with this, empathy scores are negatively correlated with insula activation only in participants with ASD during vicarious pain perception (133). In addition to autonomic hyperarousal evoked by these stimuli, it is plausible that alterations in the regulation loop between central and peripheral responses during subjective and vicarious pain experiences may be related to empathy modulation in ASD. It is also possible that difficulties in recognizing and reporting on one’s own (and others’) emotions, referred to as alexithymia, may be more responsible for observed alterations in empathy than core autistic features themselves (48, 110). For example, it has been shown that higher levels of alexithymia is related to increased arousal and diminished habituation to negative emotional stimuli (138). However, this conjecture remains unresolved (139).

The role of embodiment, e.g., the integration of sensations from one’s own bodily experience (in the present as well as in the past) in the construction of conscious perception in order to understand our own experience, and the experiences of others (140) in ASD is discussed (92). Embodied cognition is based on the same neurophysiological mechanisms which are activated during observation of stimulation applied to others as during perception by self (141–143). When participants observed someone’s body parts being injured, participants with ASD had increased activation in the *somatosensory cortex* (S1/S2) and decreased activation in the *medial prefrontal cortex* comparatively to controls (29). In addition, only participants with ASD demonstrated that S1/S2 overactivation evoked by vicarious pain was associated with lower pain pressure thresholds (e.g., higher pain sensitivity) and, paradoxically, to reduced unpleasantness rating scores. These results suggest that atypical coupling between embodiment and atypical sensory processing influences perception of vicarious pain in ASD. Consistent with these findings, reduced embodiment reaction in response to emotional stimuli is demonstrated in autism (144). Interestingly, production of an empathic response to other’s pain requires directed attention from individuals with ASD (145), which is attenuated when the individual is distracted.

The attentional demands required to detect others’ pain may partially explain discrepancies in insular cortex responses in subjects with ASD: overactivation of the insula demonstrated in Gu et al. (133) or inhibition of its activity shown in Fan et al. (29). While in the former study the images of different type (pain/no pain) were counterbalanced and randomized, Fan et al. presented stimuli in blocks of consecutive events, creating the possibility of priming the subject to the valence (pain/no pain) of the upcoming stimulus. This latter observation may be congruent with lower insula activation for sustained pain stimulation observed in Failla et al. (62). Taking into account the integrative role of the insula in the perception of the self, these observations may demonstrate alterations in upper-level integrative processes in ASD during vicarious pain perception.

In summary, results from the limited number of studies on brain reactivity to vicarious pain in ASD need to be replicated to clarify the patterns of activation of each area of the pain matrix, compared to typical activation. Previous discrepancies concerning somatosensory or insula activities during empathy for pain may arise from differences in experimental paradigms, as already described in similar studies of neurotypical development (146). Nonetheless, these studies identified potential specific alterations in brain activity, similar to those reported in studies of direct pain stimulation, especially in the (pre-)frontal brain areas. This suggests a possibility of shared origins between atypical responses to pain stimuli and difficulties in empathy (at least for pain) in ASD. According to embodiment theories, altered perception of pain in oneself may be related to anomalous reactions to signs of pain, and may be related to other socio-cognitive deviations in ASD, such as attenuated distinction between positive vs. negative emotions (124).

#### Social Touch and Down-Regulation of Pain in Autism

The social component of pain modulation must not be underestimated in the global processing of pain, particularly in ASD. The observation of someone’s pain induces a need for action in observers, an urge to provide help or reassure the person in distress. One of the most common ways to respond to someone’s pain, to reassure and calm, is through the so called “social,” or “soft,” “interpersonal,” or “affective” touch, a specific type of tactile stimulation provided by others, promoting pleasant feelings, approach-related behaviors and reductions in pain (125). It has been reported that brain correlates of soft touch-induced analgesia, included modification of pain-related activation in “nociceptive” brain areas as well as secondary (“salience”) and third-order pain perception areas, and in sub-cortical regions such as amygdala, hypothalamus, and PAG (125). Affective touch signals are conveyed to the brain *via* the specific variety of type C nerve fibers, so called C-tactile, CT fibers (127). CT fibers stimulation activates the insula, but not somatosensory areas S1 and S2 and underlies positive affective aspects of touch (147). However, these types of interactions are often reported as stressful by people with ASD, provoking unpleasant feelings or touch-aversion behavior (148).

Recent studies demonstrated that brain correlates of social touch are modified in ASD (149–151). These alterations in brain activation were correlated with the autism severity (150). Moreover, while activation of the insula and other socio–emotional brain regions were reduced, social touch evoked atypical hyper-reactivity in primary somatosensory cortex (151), consistent with increased tactile sensitivity in areas innervated by CT-fibers (30). This may explain the unpleasantness of social touch and interruption of the mechanism of soft touch-induced analgesia in autism.

Social touch promotes communication through oxytocin-dependent mechanisms (152). *Oxytocin*, a neuropeptide receiving increased attention because of its prosocial effects, has potential in social interactions improvement in autism (153, 154). The regulatory role of oxytocin on pain processing may be found on various levels from peripheral antinociception to high-level emotional pain, pain anticipation and pain memory (155). Some studies demonstrated that oxytocin treatment potentiated social brain connectivity and behavioral improvements in subjects with ASD (156, 157) and reduced heat pain intensity ratings and amygdala activation during painful stimulation (158, 159). This suggests the therapeutic potential of oxytocin in modulation of pain down-regulation by others in autism.

Formation of adequate top-down regulation of pain requires conscious appraisal of it emerging from coordinated co-activation of numerous brain areas. Decoupling between first and second order brain areas of the pain matrix (for example, under anesthesia) leads to nociceptive matrix-elicited physiological and hormonal responses (160, 161), but absence of down-regulation from upper brain areas (162). Such blunted reactions may have long-term consequences such as anxiety, pain sensitization, depression, or post-traumatic stress disorder (70, 163). In ASD, an increase in SIB (21, 28) or other-injurious (164) behavior after painful procedures, along with increased physiological and hormonal responses (165) could be considered consequences of such dysregulation.

To conclude, a body of evidence suggests atypical neuronal processing of pain in ASD. Although some recent results indicate possible alterations in nociceptors (46) or suggest possible excitatory/inhibitory imbalance in the spinal cord level (5), most of the findings focus on atypical brain processing. Together with evidence of hypoactivation in areas of the second and third order brain areas, more consistently in (pre-)frontal cortex, scientific findings support the hypothesis of a “feed-back defect” or “altered top-down regulation” in pain processing, in ASD (62). In addition, the crucial role of attentional engagement in resulting pain-evoked response may be proposed. In many studies these alterations correlated with participants’ assessment of direct pain, linking central mechanism dysfunction with atypical expression of pain in ASD. This section emphasizes the linkages between deviations in social regulation and pain perception in ASD.

***Pain perception modulation in ASD highlights.** Pain processing in the brain relies on coordinated activation of multiple brain areas. Reorganization of that response may decrease or increase levels of perceived pain. Affective and cognitive aspects of pain perception, as well as in vicarious pain experience and down-regulation of pain response by social touch seem to be modified in people with ASD. Observed alterations should be taken into account when considering the role of social reciprocity in pain regulation in autism.*

## Atypical Pain Reactions in Autism Spectrum Disorder

Emotional expressions (bodily, verbal, and facial) have an important social function in communicating one’s own feelings to others to receive adequate responses. Pain is a complex socio-communicative experience which includes biological, physiological and cognitive but also social determinants. Expressions of pain can be enhanced or suppressed by the presence of others and social context, as well as by the personal history of reassurance and support, in reference to the “social communication model of pain” (166). Displays of pain command the attention of observers and provoke reflexive behaviors that facilitate down−regulation by various mechanisms of distraction, reassurance or social referencing (36). Alternatively, individuals with autism may mask or camouflage their feelings (167), which may be associated with adverse mental health outcomes. Alterations of any of the top-down modulation steps including clear pain communication (Figure 1) may lead to aggravation of painful experience (168).

Different levels of pain communication exist. They include more or less volitionally regulated facial and body expressions, vocalizations, withdrawal from the source of the pain and also visible physiological signs of distress, like increased pupil diameter, sweating, pale skin, increased heart and respiratory rate, mediated by specific changes in hormonal and neurochemical responses (36). It is still debated (24, 27) whether atypical patterns of pain expression in ASD are mostly attributed to alterations in pain processing and regulation itself, or in the motor response realization, as alterations in motor skills are frequent in autism (169).

***Challenges of pain assessment in ASD.*** Historically, hypo-responsiveness to painful stimuli was included as one of the symptoms of ASD as “apparent indifference to pain” (19), also largely reported by clinicians and relatives of the patient (22, 25, 170). During evaluation of every-day pain reactivity, among several types of behaviors rated as “very often occurred” after a painful incident in people with ASD, some were similar to individuals without ASD (“seeking comfort,” “crying”) and some were atypical (“being difficult to distract,” “jumping around,” “agitated,” and “fidgety”; 171). However, despite the higher incidence of pain-evoking accidents (172), missing, delayed or aberrant reactions to painful stimuli are often reported in daily observation of subjects with ASD.

***Observer-rated pain assessment*** may provide a long-term profile, and thus is well-suited for chronic pain conditions. Various scales have been specifically adapted for pain assessment in populations with communication and cognitive alterations, as a function of the age and global mental functioning of the person. We can cite the short-form McGill Pain Questionnaire, SF-MPQ (49, 173); the Non-communicating Children’s Pain Checklist, NCCPC-R (174, 175), the Pre-Linguistic Behavioral Pain Reactivity Scale, PL-BPRS (21, 165); the Faces, Legs, Activity, Cry and Consolability – Revised (FLACC-R) or the Faces Pain Scale-Revised and a Numeric Rating Scale (176). These scales are focused on behavioral signs, extracted from body language and facial expression, in particular on visible signs of agitation, stress and discomfort. Importantly, in ASD many signs of emotions including stress, anxiety, discomfort, but also pain are considered as “idiosyncratic.” Thus these methods of pain evaluation in individuals with ASD, who are known to express atypical responses, should be applied with caution. Parents and observers must be familiar with the “common behavior” of the person, in order to catch the new “signal” and to recognize it as the response to pain (24, 26, 177).

***Self-assessment of pain*** gives direct, but subjective information and requires familiarization with the scales. Tools that obtain quantified self-reports of pain intensity usually allow the individual to state a number or point to a face that correlates with a number, such as the Wong-Baker Faces Pain Rating Scale (178) or the Pain-O-Meter, a plastic tool with a moveable marker (179). Self-evaluation could be difficult in an acute pain situation, even more so if the person has intellectual and conceptual difficulties and cannot follow instructions for self-reporting or has limited understanding of concepts necessary for tool utilization. Ely et al. promoted an alternate *interactive* approach to pain assessment in ASD based on individualized consideration and estimation of pain assessment methods. Utilizing the help and knowledge of parents appeared to be essential in identifying the presence of pain and accurately estimating its intensity (177). More research must be completed to advance knowledge and practice for assessing the pain of the individual with ASD. Researchers developing new tools should recruit parents for assistance.

***Global motor response.*** Based on the use of these various scales, some studies have reported a reduced behavioral reactivity to pain in children with autism, whether at home (parental assessment), in institutions or during a venipuncture (professional assessment; 165, 180, 181), but hyper-reactivity was also suggested in similar experimental conditions as well as in other clinical settings, such as during dental cleaning (182, 183). In addition to behavioral observations with traditional scales, delayed or aberrant reactions to painful stimuli are often reported in daily observations by parents and clinical reports (171). These are: lack of protective body position or withdrawal reactions (7); a wide range of deviant pain-evoked responses like: hyperactivity, paradoxical laughs, aggressiveness, stereotyped behaviors and SIB (20, 22, 165, 184); a general expression of discomfort without localization of the source of pain, as well as other variations (171).

Very few tools to capture atypical signs that are not listed in traditional scales have been tested or adapted for individuals with autism. Only one adaptative scale: the “Simplified Pain Evaluation Scale for Dyscommunicative Autism Spectrum Disorders: ESDDA” has been proposed, but it currently lacks psychometric validation (185). The development of reliable and validated assessment tools remains challenging, because of the high heterogeneity of ASD clinical features but also because pain is defined as a highly subjective experience that varies among individuals in general.

***Facial expression of pain in ASD.*** Facial expressions of pain are a crucial component of social signaling (186–188), even in non-communicative or unconscious patients (189).

Facial emotion recognition and the ability to convey emotion through facial expression has been a topic of interest in autism research for more than three decades. In people with ASD, facial expressions are often perceived as awkward or atypical for a review, see (190). The idea that people with ASD spontaneously express less frequent and shorter emotional expressions and of lower quality (less accurate and intense) than their neurotypical peers is widely accepted (191, 192), described as “amimia” in clinical reports (191). Many screening and diagnostic tools for ASD include the items: “range of facial expression” or “inappropriate facial expressions,” as one of the early signs of ASD (e.g., the Social Communication Questionnaire, Modified Checklist for Autism in Toddlers, Childhood Autism Rating Scale, Autism Diagnostic Interview).

Studies examining pain expression in autism have generally documented mixed results that have failed to provide a consensus. For example, during a venipuncture, some authors reported more facial expressions in children with ASD than in their neurotypical peers (175) whereas no differences were identified in another sample (193). To note, the existing methodologies based on manual scoring of facial expression are strongly dependent on the abilities of the observer (194–196). To minimize the impact of subjective bias and context, several studies were designed to explore facial expressions accompanying pain through standardized conditions and tools and to compare observational rating scores with physiological markers of pain perception during routine clinical procedures, such as venipuncture (165, 175, 193, 195) or dental procedures (183).

Nader et al. (175) has analyzed facial expressions in children using the most frequently used tool in traditional research on facial emotion expressions: the Child Facial Action Coding System for children (FACS) during a venipuncture. The study demonstrated that participants with ASD had greater facial response during the needle insertion phase than the children in the control group. However, other results indicated a lack of concordance between the observer and parental scores of pain evaluations (193). In addition to this, facial reactivity was compared with the motor and physiological responses (heart rate) in children with ASD, children with developmental delay and neurotypical controls.

Only children with ASD demonstrated elevated facial and motor reactivity after the end of the venipuncture, and authors indicated that the age-dependent decrement of pain-induced facial reactions was not observed in this group (193). To avoid observer-related subjectivity, automated facial emotion recognition technologies based on FACS were developed and tested in experimental settings (197–205), however, they need to be adapted to individual features of a person with ASD. To our knowledge, currently no experimental design has been proposed to test and refine algorithmic detection of pain in individuals with ASD.

***Physiological correlates of pain perception in ASD.*** The experience of pain evokes numerous physiological responses driven by the sympathetic and parasympathetic nervous system (206–208). The acute experience of pain causes an increase in heart and respiratory rate, skin conductance response, blood pressure, and pupil diameter, all features of sympathetic nervous system activation. Painful stimuli also provoke heart rate variability changes, mostly an increase in low frequency vs. high frequency ratio (209), muscle tension and a decrease in skin temperature see for review (210). Most of the existing results reported similar physiological responses in individuals with and without ASD, with regard to skin conductance responses (72) and heart rate (193). Only one study indicated increased heart rate in children with ASD compared to children without ASD, after a venipuncture (165). After venipuncture, the low-functioning autism participants demonstrated increased rates of injurious behavior directed toward others (164). Importantly, prediction models based on the integration of various physiological signals (electrodermal activity and cardiovascular activity) with motor response measured by accelerometry may be applied to anticipate the behavioral outcome in non-verbal patients with autism (211), and, potentially, to prevent negative affect and pain after manipulation.

Several limitations, related to the medical manipulation itself, should be mentioned. As venipuncture or dental care include not only painful stimulation, but also different manipulations of the subject’s hand or mouth (a tourniquet placement, numerous touches to find the vein, disinfection of the skin, or mixed sensory stimulations – lights, odors…), these procedures may generate certain level of stress. Taking in mind high unpleasantness of touch or odors for some individuals with ASD and possible impact of other psychological issues (increased anxiety, for example), these stressors may obscure the response to pain. Therefore, it would be optimal to disentangle several factors of such manipulation to characterize the specific pain-induced reactions.

To conclude, more research is needed to address the broad heterogeneity of pain expression in ASD. Experimental and clinical assessment of pain expression in ASD should be addressed by a multimodal approach, combining data from self-assessed pain, as well as the assessment of pain by an observer, with an effort to adapt and validate the traditional scales. In addition, pain reactions should be assessed *via* the automated analysis of facial expressions and body movements, as well as skin temperature and conductance; vocalizations, and other physiological signals, to create a complete profile of pain-evoked response (212, 213). Moreover, during studies that exploit the analysis of pain-evoked responses in clinical settings, efforts should be made to minimize anxiety and distress due to novelty and anticipation of pain as it may undesirably magnify pain perception in individuals with ASD (168). Paradoxically, in a recent study (175), the children who were the most reactive to pain were those who were described as the least sensitive and reactive to pain by the parents. To explain these results, the authors propose that the expression of pain differs according to the type of pain (care-related acute pain, daily acute, or chronic pains). While participants with ASD would have atypical or reduced behaviors in an everyday situation, they would express themselves in an exaggerated way during a care situation (175). Thus, in the future, preference should be given to non-invasive or more naturalistic methods with minimum manipulations or novelty.

To achieve this goal, new technologies provide some innovative approaches (190, 211). The use of wearable devices allows to assess a range of physiological and behavioral reactions in individuals with ASD (214); however, to date, these studies were not aimed to evaluate pain-evoked responses. Neuro-feedback based strategies, which would join individual neurophysiological correlates of pain experience with simultaneously recorded expressions of pain, may provide a pathway for people with ASD to express their feelings and solicit reflexive behaviors from social partners.

***Atypical pain reactions in ASD highlights.** Production of an adaptive reaction to a painful event is indispensable to receive adequate social support. In individuals with ASD, reactivity to pain is often changed, leading to the absence of proper pain management and corruption of pain cycle functioning. The dissociation between central and autonomic responses to pain may be a cause of alterations of pain processing in ASD. The need for an integrative evaluation of pain responses utilizing modern real-life technologies is necessary to address the individualized needs of each patient.*

## Mechanisms and Perspectives

### Summary of Common Mechanisms, Involved in Both Autism Spectrum Disorder and Pain Regulation

Many neurochemical pathways involved in determining ASD are also known to participate to pain processing in typical development (see; 215). The pain regulation pathways are predominantly noradrenergic and serotonergic, but implicate other neuromodulators including dopamine and opioids, as reviewed in Yam et al. (35). Here we will discuss the role of some neurotransmitters in ASD in relation to the regulation of pain.

#### Opioids and Gut Hormones

Endorphins are important components of endogenous pain control (35, 208, 216). Peripherally, beta-endorphins produce analgesia by binding to opioid receptors. At the level of CNS, mu-opioid receptors block pain processing and activate descending pain control circuits including the amygdala and PAG (216). The “Brain Opioid Theory of Social Attachment” (BOTSA), in which social and affiliative behaviors were proposed to depend on endogenous opioid peptide levels, has been based on similarities between social attachment and drug addiction (217). Many symptoms of ASD seems to resemble behaviors induced in animals or humans by opioid administration, including: reduced socialization, repetitive stereotypies, motor hyperactivity and especially insensitivity to pain. This has led to the *neurochemical opioid hypothesis* in ASD, based on evidence from animal models and human studies and from clinical trials with opioid antagonists (naloxone and naltrexone), in the early 90s. Blood and cerebrospinal fluid studies have reported that beta-endorphin levels were altered in individuals with ASD (218–222). In children with ASD, compared to neurotypical children, increased opioid levels were found after exposure to painful manipulation (venipuncture), which were correlated with autism severity (165). However, these results failed to be replicated, that may been ascribed to measures biases (165, 219–222).

After years of disinterest, the role of opioids in ASD could be updated. In light of recent views on the opioid antagonists effect (223) and findings on modifications in particular opioid receptors in subjects with ASD (224), imbalance in the opioid system may be proposed as one of the mechanisms of pain sensitization, hyperalgesia and increasing of pain vulnerability (225, 226). It has been hypothesized that chronic pain conditions in individuals with ASD could be responsible for SIB, which would have a calming effect through opioid release (24). SIB may be related both to enhanced expressions of pain (227), and to reduced pain sensitivity (228). In neurodevelopmental disorders, SIB has been proposed to represent a nociceptive coping behavior, mediated by altered nociception, allodynia or hyperalgesia and neuroinflammatory mechanisms similar to those in chronic neuropathic pain disorders (229). In a recent review of 10 clinical trials testing opioid antagonists and specific drugs in children with ASD, the authors concluded that only in a sub-group with elevated endorphin levels, naltrexone was effective in improving SIB, hyperactivity and restlessness (223).

Endogenous opioids are implicated not only in pain downregulation, but also play a role in many high-level processes like regulation of social behavior. Thus, imbalance of opioid signaling in ASD may be implicated in altered pain perception and SIB as an internal pathophysiological mechanism of opioid tone regulation, but also may contribute to social symptoms. However, the nature of the relationship between self-harm, endogenous opioids and pain processing is not clear yet.

An alternative supposition has been formulated by Jonhson et al. (230, 231), elaborating the links between opioids and ASD. The authors proposed that autism may be related to a genetic predisposition in sub-groups of individuals, triggered by administration of exogenous opioid hormone/medication at birth or during labor that interferes with the natural fetal opioid balance. However, this hypothesis hasn’t been documented in a large cohort of babies born to opioid-addicted mothers (232). To note, pre-conception opioid prescription was associated with 2.43 times more for the odds of ASD compared to mothers without prescriptions (233).

Recently, the relation between opioids and gastrointestinal disorders, which is often comorbid in autism, has been discussed (234–236). The gastrointestinal tract problems in ASD include impairments in bowel mucosa function, selective permeability, gut immune response, potentially creating a source of chronic irritation and visceral pain in these patients (237). Increased gastrointestinal tract symptoms are significantly associated with post-stress cortisol concentrations in ASD (238). Gut hormones may play an important role in such relations (239). Among them, ghrelin, a peptide hormone regulating appetitive behavior, reward, stress and anxiety response (240) is decreased in children with autism (241). Ghrelin may also be involved in pain modulation, as central ghrelin injections increased beta endorphin concentrations in PAG and reduced behavioral pain responses (242). Taken together, these observations underpin possible interactions between co-occurrence of chronic pain, SIB and gut dysfunctions, pointing toward a role of endogenous opioids and stress hormones in dysregulated pain perception in ASD.

#### The Gamma-Aminobutyric Acid Role

The excitatory-inhibitory misbalance in the neuronal system Is implicated in the pathophysiology of pain (243, 244). Gamma-aminobutyric acid (GABA) is known as the main inhibitory neurotransmitter in the adult brain. However, during prenatal stage, GABAergic regulation possesses excitatory role (245, 246). In typical development, GABAergic system switches from excitatory to inhibitory function. The *“GABA” hypothesis* suggests that early dysfunction of the GABAergic signaling disables normal excitatory/inhibitory switch and is responsible for ASD-related symptoms (247–250). This suggestion is in line with model of “developmental heterochrony,” according to which, autism-related traits may be explained by delays or truncation of typical development (251). In support of this, various studies in ASD have reported reduced GABA concentration, specifically in visual, auditory, somatosensory areas, both in children and adults (252–254).

Recent findings demonstrated that lack of GABA transmission *via* deficiency in long-term potentiation and learning mechanisms impacts the ability of *the amygdala* to form fear memory (255) and may be responsible for autistic-like features. Improvement in ASD symptoms related to Bumetanide therapy (a drug promoting of GABA shift from excitation toward inhibition) in young children (<6 years old) was associated with normalization of GABA/Glutamate ratios specifically in the insula cortex (256). This may relate GABA dysfunction and the role of negative affect in socio-emotional learning of pain perception. Two other recent studies analyzed the relationship between GABA levels in several brain regions and multiple aspects of sensory processing alterations in ASD. The first study revealed that GABA concentration in the sensorimotor cortex of adults with ASD was lower than in neurotypical adults. In addition, GABA levels were positively correlated with self-reported tactile hypersensitivity in adults with ASD, but not in neurotypical adults (257). In line with these findings, another study in adults with ASD revealed a negative association between left ventral pre-motor cortex GABA concentration and the severity of sensory hyper-responsiveness scores on the Adolescent/Adult Sensory Profile (258). To our knowledge, no study has examined whether GABA concentrations in the brain were associated with altered pain experiences in people with ASD. Future work should evaluate GABA levels in the brain pain matrix as a significant biomarker and therapeutic target for autistic sensory processing disorder and specifically pain processing.

#### The Role of the Endocannabinoid System

In direct relation with GABA neurotransmission, cannabinoid receptors are present in brain and peripheral neurons, the vagus nerve, gastrointestinal lining, immune cells and skin (259). The ECS participates in brain development, synaptic plasticity, sensory processing and integration, stress regulation and neuromodulation (260). This system modulates social interactions, anxiety and social reward (261). In the context of dysregulation of pain perception, cannabinoid receptors have also been found to play a role in sensory transmission and pain integration (262). Endocannabinoid system dysregulation has been proposed as a pathophysiological mechanism of autism (263, 264). Decreased levels of endocannabinoids were found in children with ASD (265); see for systematic review (266, 267) to link various symptoms in ASD (inflammation, microglia dysfunction, and social symptoms; 268). Three recent papers published by the same team (269–271) focused on the modulation of the brain’s excitatory and inhibitory systems in adults with ASD and neurotypical controls, after a single dose of 600 mg of cannabinoids. This treatment increased levels of GABA *in the dorsomedial prefrontal cortex* of controls, but decreased GABA in that region of participants with ASD. In light of these findings, new avenues of therapeutic intervention in the treatment of autism have emerged, on one hand from empirical experience of patients and members of their families, and on the other hand, leveraging genetic and metabolic research. In relation to the above-mentioned gut-brain axis dysfunction in ASD, the potential for the endocannabinoids in alleviating gastrointestinal (272) and behavioral disorders in autism (263, 273–275) along with pain management (262) should be explored. However, the precise mechanism of action of cannabinoids in ASD is far from complete. For example, the two main active components, tetrahydrocannabinol and cannabidiol, interact divergently with cannabinoid receptors. The former activates the receptors, whereas the latter seems to block them (260). However, both components were found to improve behaviors in autism (273, 276), indicating needs to future investigations.

Thus, numerous alterations of neurochemical pathways implicated in autism (Table 1) may provide a rich background for integrating numerous behavioral and neurophysiological alterations documented in pain perception, as well as in the development of strategies for pain treatment.

**TABLE 1:** Summary and discussion of mechanisms involved in both ASD and pain dysregulation.

Mechanism	Description	Justification	Criticism
Neurochemical modulations: *Opioid system dysregulation*	As endogenous opioids are an important component of pain downregulation, modifications of opioid signaling in ASD may cause altered pain perception	Increased levels of opioids observed in ASD after painful manipulation; opioid antagonists seems to alleviate ASD symptoms opioid receptor dysfunction found in ASD	Elevated opioid levels were not confirmed in subsequent studies
Neurochemical modulations: *Interrupted GABA switch*	Pain perception is altered by excitatory-inhibitory imbalance in the developing nervous system	Various studies have reported reduced GABA concentrations in perceptive areas in ASD	No studies demonstrate pain alleviation in ASD with GABA agonists/antagonists or any relation between GABA brain levels and pain
Neurochemical modulations*:* *Endocannabinoid dysregulation*	Endocannabinoid system dysregulation has been proposed as a pathophysiological mechanism of autism	ECS participates in sensory transmission and pain integration. Cannabinoids alleviate autistic behavior symptoms	No studies demonstrate relation between ECS brain levels and pain perception in ASD
Pain processing modulations: *Global sensory processing alteration*	Atypical pain perception in autism due to global alterations of sensory perception mechanisms (peripheral or central)	Sensory alterations are present in the majority of cases and are commonly recognized as diagnostic criteria in ASD; modifications in the structure of nociceptive sensory endings were reported in ASD; consistent with these findings, allodynia, paradoxical heat sensations and alterations in C-tactile soft touch perception are documented in autism	Intact, hypo-, and hypersensitivity may be observed depending on methodology. Pain-related responses in population with ASD are heterogeneous. Sensory processing patterns are known to change over time, depending on the context in which they are observed
Brain pain processing modulations: *Altered pain processing and response regulation*	*pain cycle imbalance in ASD:* reduced engagement of the pain matrix and in-adequate physiological responses disable adequate cognitive and emotional regulation of pain perception	In ASD, decreased response of the pain matrix might be a result of reorganization of pain processing pathways. Altered interoceptive abilities and self-consciousness in ASD do not allow cognitive appraisal of all aspects of pain and thus makes impossible proper down-regulation, which may lead to inadequate pain-related responses, like self-injury behavior	Descending noxious controls have been found to be normal in autism Little data is available on brain processing of pain in ASD
Modulation of both pain expression and social feed-back: *Socio-communicational issues*	Social and cognitive complications in ASD prevent communication about perceived pain and requests for help	According to theories regarding the role of cognitive and emotional control of pain, altered embodiment of emotions in ASD and atypical pain expressions in ASD may lead to atypical development of recruiting the help of others in managing pain in ASD	In spite of some alterations in brain processing. perception of vicarious pain seems to be normal in ASD
			

#### Global Sensory Processing Alterations

Sensory atypicalities are recognized as diagnostic criteria in ASD (19). 90% of individuals with ASD have atypical sensory experiences and regulation, described as both hyper- and hypo-reactivity, with altered responses to tactile or auditory stimulations (75). This topic has been the focus of intensive research over the last 10 years, including studies on pain perception, leading to the “*hypothesis of sensory dysfunction” as a* cause of altered pain sensitivity in ASD (14, 30, 32). These anomalies affect all the sensory channels of the child and the adult (see section “Initial Stages of Pain Signaling: From Nociceptors to the Central Axis”) and complicate the integration and cognitive processing of stimuli, making each pain percept unique and poorly integrated in a general concept of the self.

#### Altered Central Pain Processing and Modified Regulation of Pain Responses

**FIGURE 3:**
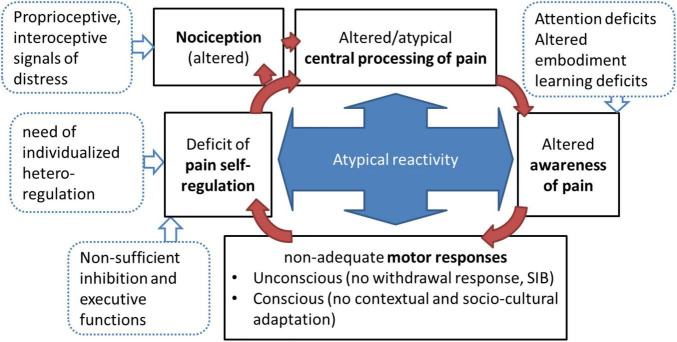
The pain cycle in ASD with possible impacts from suggested mechanisms, modified integrated model, adapted from Dubois et al. (23), Rattaz et al. (24), and (277).

A growing body of evidence delineating alterations of central (both cortical and subcortical) and peripheral pain transduction, transmission and processing are challenging the idea of impaired pain processing due to a single factor, and calls for an integrative model. To summarize the findings regarding the central brain responses and altered pain reactivity in ASD (see section “Neurophysiological Processing of Pain in Autism Spectrum Disorder”), we propose the integrated model of the pain cycle in autism (Figure 3). Within this framework, evidence for altered self-awareness and interoception, and reciprocal relations between altered top-down regulation of pain processing and pain coping in autism may lead to inadequate and deviant pain reactivity. By analogy with other chronic pain conditions, we speculate that altered pain processing in the brain of individuals within the spectrum results from reorganization of pain pathways. This suggestion is supported by recent findings from neuroimaging studies (62, 71, 95). However, more research is needed to delineate causal dependencies between these diverse processes, and to demonstrate how pre-conscious nociception relates to conscious pain perception in subjects with autism, given their self-awareness and interoceptive abilities. Although the basic processes of pain down-regulation seem to be unchanged, the capacity for conscious appraisal of pain and the communication of pain to others might be limited in ASD, especially in early life, when imbalances in physiological and hormonal responses lead to dysregulation of pain management, and probably to long-term consequences like increases in anxiety and depression (168).

#### Socio-Communicative Issues

Communicational model of pain highlights the social role of pain expression and intrapersonal reciprocity in down-regulation of pain (277). Observed dissociation between enhanced physiological and biological stress responses and behavioral reactions in autism (28, 165) suggest that, at least for some individuals, the neurochemical and brain pathways are preserved but pain communication is altered (8, 177, 181). Based on the various findings reported above (see section “Atypical Pain Reactions in Autism Spectrum Disorder”) atypical motor response and altered reciprocity may inhibit the observer’s empathy, raising a “double-empathy issue” (278) that was highlighted in recent publications (23, 24). As in most cases, people with ASD have no intellectual disability (279) and may use alternative strategies of social adaptation (280), the potential of specific cognitive-behavioral therapy (281) for pain understanding in autism should be explored.

### Perspectives

Do core autism symptoms (sensorial and social) ***initially*** cause altered pain perception and expression, which are then worsened by comorbidities and atypical social modulation experiences? Or does pain processing in both neurotypical and ASD individuals share ***common vulnerabilities*** that lead to association of ASD symptoms and pain dysregulation? Would it be possible to compensate or prevent alteration of the pain modulation as early as ASD is diagnosed? General heritability of ASD is approximately 80% (282). Among the numerous genes associated with ASD, some have also recently been implicated in pain processing (283, 284). These genes are involved in various cytomolecular mechanisms controlling of C-fiber excitability thresholds, or glutamate pathways (27) and induce alteration in pain processing, in both animal and human models (5) if muted. However, findings in animal models of autism report wide divergence of pain perception and responses (27), pointing out the possible impact of epigenetic factors on relevant genetic alterations of ASD. To note, most animal models are monogenic mutation models, in contrast with human. Additional research is needed to better understand the common genetic and biomolecular pathways (283, 284), and propose innovative and preventative therapeutics.

Pain processing develops with age, starting during gestation, and matures with social experiences throughout life (285, 286). Given that ASD is considered a developmental condition, future research need to address this developmental aspect, proposing longitudinal studies, including early therapeutics and preventative strategies.

Social development interacts with pain management. Positive social experiences impact pain modulation and may alleviate pain perception. Oppositely, negative social experiences as isolation, bullying, and social rejection among others, may aggravate perceived pain. Such “psychological” pain itself produces similar brain response in pain matrix as does physical pain (287, 288) and have been associated with SIB in ASD (289). That bidirectional aspect of social interactions on pain perception and expression in ASD should be more explored.

***Mechanisms and perspectives highlights.** Numerous mechanisms involved in ASD pathogenesis have been proposed to explain the alterations in pain processing. Among them, alterations in neurotransmitters and neuropeptides (opioids, endocannabinoids, GABA), in global sensory processing, in brain response, together with socio-communicational issues are thought to be associated differently in individuals with ASD contributing to both autism core symptoms and pain dysregulation. In an attempt to integrate these mechanisms, we propose our all-inclusive model of the pain cycle in autism. However, a number of open questions still remains, requiring an integrative approach to resolving the paradox of pain in ASD.*

## Conclusion

The research of pain in ASD is complex. Recent findings in alterations of brain pathways, summarized as an integrated model of the pain cycle in autism (Figure 3), suggest more than nociceptor and neuronal dysfunctions and implicate altered self-awareness and interoception, which interact with top-down regulatory pathways for processing and coping with pain. Both peripheral and central deviations in pain signal processing are documented in autism. The high variability in pain-related responses in this population makes the group-based approach challenging. To date, other- and self-rated pain assessments aren’t sufficient to characterize the specificity of pain-related processes in autism and to drive interventions. New technologies, already used in emotion recognition research in ASD, joined with continuous physiological activity screening, may provide more quantitative and integrative approaches to specify what is atypical in expressions of pain in people with ASD. Future directions may address the role of these various alterations at different stages of pain signaling and regulation in ASD symptomatology and may yield promising candidates for global therapy, social improvements and behavioral amelioration. The importance of adequate, objective and multiple approaches for the assessment of pain in individuals with autism is essential not only for health outcomes but also to prevent the worsening of social disorders. A better understanding of the complexity and individuality of pain regulation may resolve the paradox of pain in autism, leading to the development of individualized pain management strategies, and with novel therapeutic approaches.

## Author Contributions

OB: conceptualization and writing – original draft. VB: visualization and writing – review and editing. AP, MB, and NM: writing – review and editing. J-RC: funding acquisition, supervision, and writing – review and editing. AA: funding acquisition, supervision, conceptualization, and writing – review and editing. All authors contributed to the article and approved the submitted version.

## Conflict of Interest

The authors declare that the research was conducted in the absence of any commercial or financial relationships that could be construed as a potential conflict of interest.

## Publisher’s Note

All claims expressed in this article are solely those of the authors and do not necessarily represent those of their affiliated organizations, or those of the publisher, the editors and the reviewers. Any product that may be evaluated in this article, or claim that may be made by its manufacturer, is not guaranteed or endorsed by the publisher.
