# Supplementary material for: The Current View on the Paradox of Pain in Autism Spectrum Disorders
Source: Front Psychiatry. 2022 Jul 22;13:910824. doi: 10.3389/fpsyt.2022.910824 (PMC9352888; doi:10.3389/fpsyt.2022.910824)
Supplement: Supplementary file 1 [file Table_1.DOCX]

SUPPLEMENTARY MATERIALS for the paper

**The current view on THE PARADOX OF PAIN IN AUTISM SPECTRUM DISORDERS**

Olena V BOGDANOVA^1#^, Volodymyr B BOGDANOV^2^, Adrien PIZANO^3^, Manuel BOUVARD^1,3^, Jean-Rene CAZALETS^1^, Nicholas MELLEN^4^, Anouck AMESTOY^1,3^

| **Supplementary table 1. General pathways of pain perception and regulation** |
| --- |
| **Nociception** (1–3). |
| **Nociceptors: activation of** the first order sensory neurons with two different types of axons*:   - rapid, myelinated ***Aδ (A-delta)*** fiber axons; acute pain transmission - slow, ***non-myelinated C fiber*** axons with slow adaptation, pain sensitization and hyperalgesia   *recently ultra-fast myelinated, type A pain sensitive neurons were discovered in humans |
| **From nociceptors to the brain** (1,2,4–7) |
| First-order neurons connect to the second order cells on the spinal cord. There are two main pain ascending pathways (mostly contralateral):   - ***spinothalamo-cortical pathway*** that processes the discriminative aspects of pain (to the thalamus) - ***spinoparabrachial-amygdala(-limbic) pathway*** that processes the affective-motivational aspects of pain (to PeriAqueductal Gray (PAG) matter of the midbrain, the lateral parabrachial area in the pons, amygdala and others nuclei) |
| **The high-level processing of pain**  (8–17) |
| Second-order neurons connect to third-order neurons, which project to upper brain areas:  ***“the pain matrix”:*** the *primary somatosensory cortex* (*S1):* direct somatotopic input from the thalamus; *the secondary somatosensory cortex (S2), the insula, cingulate, orbitofrontal and prefrontal cortices, and the amygdala:*   - nociceptive information simultaneously reaches sensory, motor and limbic regions of the brain, with a specific hierarchy in their activation:   ***-first order brain area: ‘the nociceptive matrix’:*** sensory (*the primary somatosensory cortex*, *the* *posterior insula, adjacent suprasylvian operculum, and posterior parietal regions)* and motor-orienting *(the posterior midcingulate and supplementary motor area)*   - - bodily pain perception and rapid pre-motor and orienting response; manipulation may induce pain or selective loss of nociception, may be unconsciously activated   ***-second order brain area: ‘the salience matrix’:*** *posterior parietal, prefrontal and anterior insular areas:*   - - transition from the ‘nociceptive matrix’ activation to conscious pain perception   ***-third-order brain areas:*** *anterolateral and orbitofrontal, ventral tegmental and perigenual /limbic* areas:   - - initial pain experience modified by cognitive, affective and motivational factors   - impact of autobiographical memory and self-reference integration, s*elf-consciousness,* proper pain perception and localization, emotional components of pain perception, pain expectation and expression |
| **Chronic pain** (18) |
| Pain chronification leads to increase in connectivity between somatosensory brain areas and the self-representational Default Mode Network brain regions (*the medial prefrontal cortex*, *posterior cingulate cortex*, *inferior parietal cortex* and *precuneus*)(19–21), misbalance between pain pathways and embodiment of pain (18). |
| **Pain regulation** (1,4,6,22,23) |
| In the ***corticofugal pathway*** the pain-associated brain areas project descending axons towards the rostral ventromedial medulla, the dorsolateral pontomesencephalic tegmentum, and PAG towards the *dorsal horn of spinal cord.* Pain down-regulation may be inhibitory and excitatory:   - Descending projections from *S1, cingulate and insular* cortex facilitate sensory transmission, provoking pain hypersensitivity and/or maintaining chronic pain - *ventro-medial prefrontal cortex,* possesses antinociceptive effects via down-regulation of other pain-sensitive brain areas and modulates of ascending pain signals via *PAG*-dependent pathway   ***PAG*** receives inputs from thalamus, hypothalamus and cortex and sends direct and indirect projections to *reticular formation, several structures of midbrain, thalamus, and ventral tegmental area and substantia nigra*. Stimulation of PAG produces profound analgesia by anti-nociceptor inputs to *nucleus raphe magnus*, which projecting to the spinal cord and block there pain stimuli transmission, but it may also facilitate nociceptive signal transduction. |

| **Supplementary table 2. Abbreviations used in the paper text** | |
| --- | --- |
| ASD | Autism spectrum disorder |
| CFCS | the Child Facial Action Coding System for children |
| CNS | central nervous system |
| CPM | Conditioned Pain modulation |
| CT fibers | C-tactile fibers |
| DMN | default mode network |
| DNIC | Diffuse Noxious Inhibitory Controls |
| ECS | Endocannabinoid system |
| ESDDA | Simplified Pain Evaluation Scale for Dyscommunicative Autism Spectrum Disorders |
| FLACC-R | Faces, Legs, Activity, Cry and Consolability – Revised |
| GABA | Gamma-aminobutyric acid |
| HRV | heart rate variability |
| MNS | Mirror Neuron System |
| NCCPC-R | Noncommunicating Children’s Pain Checklist |
| PAG | PeriAqueductal Gray |
| PL-BPRS | Pre-Linguistic Behavioral Pain Reactivity Scale |
| SF-MPQ | short-form McGill Pain Questionnaire |
| SIB | Self-injury behaviour |
| S1 | the primary somatosensory cortex |
| S2 | the secondary somatosensory cortex |

***REFERENCES***

1. Bourne S, Machado AG, Nagel SJ. Basic anatomy and physiology of pain pathways. *Neurosurg Clin N Am* (2014) **25**:629–638. doi: 10.1016/j.nec.2014.06.001

2. Yam MF, Loh YC, Tan CS, Adam SK, Manan NA, Basir R. General Pathways of Pain Sensation and the Major Neurotransmitters Involved in Pain Regulation. *Int J Mol Sci* (2018) **19**: doi: 10.3390/ijms19082164

3. Nagi SS, Marshall AG, Makdani A, Jarocka E, Liljencrantz J, Ridderström M, Shaikh S, O’Neill F, Saade D, Donkervoort S, et al. An ultrafast system for signaling mechanical pain in human skin. *Sci Adv* (2019) **5**: doi: 10.1126/sciadv.aaw1297

4. Todd AJ, Wang F. Central Nervous System Pain Pathways. *Oxf Handb Neurobiol Pain* (2020) doi: 10.1093/oxfordhb/9780190860509.013.5

5. Willis WD, Westlund KN. Neuroanatomy of the pain system and of the pathways that modulate pain. *J Clin Neurophysiol Off Publ Am Electroencephalogr Soc* (1997) **14**:2–31. doi: 10.1097/00004691-199701000-00002

6. Steeds CE. The anatomy and physiology of pain. *Surg - Oxf Int Ed* (2016) **34**:55–59. doi: 10.1016/j.mpsur.2015.11.005

7. Anderson WS, Ohara S, Lawson HC, Treede R-D, Lenz FA. “Plasticity of pain-related neuronal activity in the human thalamus.,” In: Møller AR, editor. *Progress in Brain Research*. Reprogramming of the Brain. Elsevier (2006). p. 353–364 doi: 10.1016/S0079-6123(06)57021-9

8. Lenz FA, Weiss N, Ohara S, Lawson C, Greenspan JD. “Chapter 6 The role of the thalamus in pain.,” In: Hallett M, Phillips LH, Schomer DL, Massey JM, editors. *Supplements to Clinical Neurophysiology*. Advances in Clinical Neurophysiology. Elsevier (2004). p. 50–61 doi: 10.1016/S1567-424X(09)70342-3

9. Bastuji H, Frot M, Perchet C, Magnin M, Garcia‐Larrea L. Pain networks from the inside: Spatiotemporal analysis of brain responses leading from nociception to conscious perception. *Hum Brain Mapp* (2016) **37**:4301–4315. doi: https://doi.org/10.1002/hbm.23310

10. Mazzola L, Isnard J, Peyron R, Mauguière F. Stimulation of the human cortex and the experience of pain: Wilder Penfield’s observations revisited. *Brain* (2012) **135**:631–640. doi: 10.1093/brain/awr265

11. Garcia-Larrea L. Insights gained into pain processing from patients with focal brain lesions. *Neurosci Lett* (2012) **520**:188–191. doi: 10.1016/j.neulet.2012.05.007

12. Kassubek J, Juengling FD, Els T, Spreer J, Herpers M, Krause T, Moser E, Lücking CH. Activation of a residual cortical network during painful stimulation in long-term postanoxic vegetative state: a 15O–H2O PET study. *J Neurol Sci* (2003) **212**:85–91. doi: 10.1016/S0022-510X(03)00106-0

13. Garcia-Larrea L, Bastuji H. Pain and consciousness. *Prog Neuropsychopharmacol Biol Psychiatry* (2018) **87**:193–199. doi: 10.1016/j.pnpbp.2017.10.007

14. Garcia-Larrea L, Peyron R. Pain matrices and neuropathic pain matrices: a review. *Pain* (2013) **154 Suppl 1**:S29-43. doi: 10.1016/j.pain.2013.09.001

15. Damasio A. Feelings of emotion and the self. *Ann N Y Acad Sci* (2003) **1001**:253–261. doi: 10.1196/annals.1279.014

16. Coutelle R, Berna F, Danion J-M. La mémoire autobiographique et le self dans les troubles du spectre autistique sans déficience intellectuelle (TSASDI) à l’âge adulte. *Ann Méd-Psychol Rev Psychiatr* (2017) **175**:630–635. doi: 10.1016/j.amp.2016.03.017

17. Keromnes G, Chokron S, Celume M-P, Berthoz A, Botbol M, Canitano R, Du Boisgueheneuc F, Jaafari N, Lavenne-Collot N, Martin B, et al. Exploring Self-Consciousness From Self- and Other-Image Recognition in the Mirror: Concepts and Evaluation. *Front Psychol* (2019) **10**: doi: 10.3389/fpsyg.2019.00719

18. De Ridder D, Vanneste S, Smith M, Adhia D. Pain and the Triple Network Model. *Front Neurol* (2022) **13**:757241. doi: 10.3389/fneur.2022.757241

19. Baliki MN, Geha PY, Apkarian AV, Chialvo DR. Beyond Feeling: Chronic Pain Hurts the Brain, Disrupting the Default-Mode Network Dynamics. *J Neurosci* (2008) **28**:1398–1403. doi: 10.1523/JNEUROSCI.4123-07.2008

20. Baliki MN, Mansour AR, Baria AT, Apkarian AV. Functional reorganization of the default mode network across chronic pain conditions. *PloS One* (2014) **9**:e106133. doi: 10.1371/journal.pone.0106133

21. Alshelh Z, Marciszewski KK, Akhter R, Di Pietro F, Mills EP, Vickers ER, Peck CC, Murray GM, Henderson LA. Disruption of default mode network dynamics in acute and chronic pain states. *NeuroImage Clin* (2017) **17**:222–231. doi: 10.1016/j.nicl.2017.10.019

22. Ong W-Y, Stohler CS, Herr DR. Role of the Prefrontal Cortex in Pain Processing. *Mol Neurobiol* (2019) **56**:1137–1166. doi: 10.1007/s12035-018-1130-9

23. Cameron AA, Khan IA, Westlund KN, Cliffer KD, Willis WD. The efferent projections of the periaqueductal gray in the rat: a Phaseolus vulgaris-leucoagglutinin study. I. Ascending projections. *J Comp Neurol* (1995) **351**:568–584. doi: 10.1002/cne.903510407
